# Vitamin D Intoxication and Nephrocalcinosis in a Young Breastfed Infant

**DOI:** 10.1155/2021/3286274

**Published:** 2021-07-30

**Authors:** Abdullah Al-Kandari, Hussain Sadeq, Rita Alfattal, Maryam AlRashid, Mayra Alsaeid

**Affiliations:** ^1^Department of Pediatrics, Al-Amiri Hospital, General Pediatrics Unit, Kuwait city, Kuwait; ^2^Department of Pediatrics, Al-Amiri Hospital, Pediatric Endocrine Unit, Kuwait city, Kuwait; ^3^Department of Pediatrics, Faculty of Medicine, Kuwait University, Kuwait city, Kuwait

## Abstract

Numerous studies were performed assessing the benefits and side effects of vitamin D. Vitamin D helps in regulating the calcium and phosphate metabolism leading to a healthy mineral and bone development. Vitamin D intoxication is an uncommon event that leads to hypercalcemia which can be associated with both immediate and late morbidities that can cause severe renal complications. Here, we present a case of a 4-month-old girl with a history of decreased feed and activity due hypercalcemia and high vitamin D level, which led to nephrocalcinosis. The patient received IV fluids, IV diuretics, methylprednisolone, and bisphosphonate in order to normalize the calcium level in blood. With clear verbal and written instructions for the dosage and administration of vitamin D supplements, as well as clear warnings of the potential risks of overdose, vitamin D intoxication could be an easily avoidable condition.

## 1. Introduction

In the past decade, several studies focusing on the benefits of vitamin D have been reported [1–5]. Vitamin D is an essential hormone in the human body that regulates calcium homeostasis and prevents the development of rickets [6–8]. Vitamin D intoxication is an uncommon event that leads to hypercalcemia, which is associated with both immediate and late morbidities that can cause severe renal complications [9–15]. Recently, there have been several case reports of vitamin D toxicity in very young infants due to vitamin D overdose [10, 12]. The present case study reports an infant who developed acute hypercalcemia and nephrocalcinosis after prolonged administration of an overdose of over-the-counter vitamin D supplements by the mother. Over-the-counter drugs are safe when used properly as directed by the pharmacist; however, misuse or miscommunication can lead to a rare devastating consequence, such as our report.

## 2. Case Presentation

A 4-month-old girl was brought to the emergency department with a history of lethargy, decreased feeding, and constipation for more than a month. On examination, the patient exhibited signs of moderate dehydration such as dry skin, poor skin turgor, and decreased urine output although initial vital signs were within normal limit (heart rate was 122 beats per minute, respiratory rate was 38 respiration per minute, and blood pressure was 90/60 mmHg). Routine investigations performed in the emergency department identified a high corrected calcium level (5.60 mmol/L; normal range: 2.25–2.75). Upon further questioning of the parents, it became apparent that the girl had been receiving an excessive dose of vitamin D supplementation. The patient had been receiving a full 1 mL dropper of an over-the-counter vitamin D supplement (1 mL dose equal to 5000 IU) three times a day on a daily basis, for a one-month period, instead of the intended dose of three drops once per day (three drops dose equals to 400 IU). Parents are highly educated with excellent socioeconomic status; however, miscommunication with the pharmacist regarding the dosage led to this mistake. This unfortunate event led the patient to present with vitamin D intoxication, with the blood test for vitamin 25-hydroxyvitamin D (25(OH)D) levels showing a level of 350 nmol/L (critical level: >250 nmol/L).

The infant was admitted to a general pediatric ward for further management of calcium and vitamin D levels. She was put on intravenous (IV) fluids containing normal saline and dextrose at rate of 28 ml/hour, which equals to 150 ml/kg/day. She also received 1 mg/kg furosemide IV divided into three doses per day along with the 3-day IV course of 0.3 mg/kg methylprednisolone once daily. Furthermore, the patient was fed with low calcium formula milk (Nutricia™ Kindergen).

**Figure 1:**
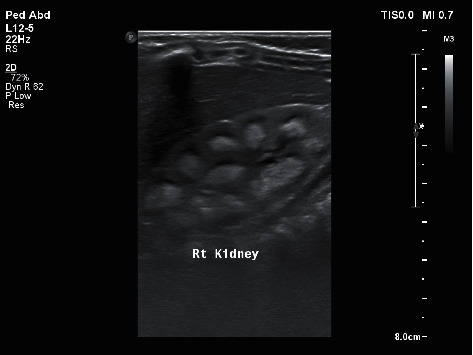
Ultrasound image of patient's right kidney showing echogenic medullary pyramids suggestive of nephrocalcinosis.

As part of the routine assessment of hypercalcemia and vitamin D toxicity, various laboratory and radiological investigations were performed to assess the extent of the toxicity on the patient's renal system. Both serum creatinine and urea were normal upon admission, 32 umol/L (normal range: 11–34 umol/L) and 5.9 mmol/L (normal range: 1.8–6.4 mmol/L), respectively. Urine analysis was performed as well and showed no abnormal parameters. However, urine calcium (mmol/L) to urine creatinine (mmol/L) ratio was significantly abnormal (6.17; normal range: 0.08–0.57). Additionally, an abdominal ultrasound was performed, showing evidence of nephrocalcinosis (Figure 1).

Furosemide (1 mg/kg/dose three times per day) was replaced with thiazide (1 mg/kg/dose twice per day) on day 3 of admission, and potassium citrate (1 mmol dose three times per day) was administered to reduce the risk of exacerbating the existing nephrocalcinosis.

The rate of reduction of the corrected calcium level was unsatisfactory, as on day 4 of admission, the level remained high at 4.17 mmol/L. At this point, bisphosphonate therapy was initiated, with two doses given (first dose of 0.5 mg/kg and second dose of 1 mg/kg).

On day 9 of admission, the corrected calcium level was 3.44 mmol/L, and the clinical condition of the patient had improved in terms of feeding and physical activity level. At the point of discharge after 10 days in the hospital, the patient's corrected calcium level had reduced to 2.96 mmol/L. The patient was discharged with a prescription of low calcium milk formula and potassium citrate (0.5 mmol three times per day). Furthermore, follow-up appointments with an endocrinologist and nephrologist were also given. Summary of daily laboratory results is gathered and presented in Table 1.

## 3. Discussion

Although breastfeeding remains the best source of infant nutrition, breast milk can lack adequate amounts of vitamin D [10, 16]. The American Academy of Pediatrics (AAP) recommends the provision of vitamin D supplements to any breastfed infant to avoid the risk of developing nutritional vitamin D deficiency, also known as nutritional rickets [10]. Guidelines issued by the AAP and the National Academy of Sciences indicate that all breastfeeding and nonbreastfeeding infants who drink <500 ml per day should receive 20 IU of vitamin D supplement per day [17]. Over-the-counter vitamin D supplements are easily and readily available [18]. The inappropriate administration of high doses of vitamin D in infants are typically provided by families for complaints such as delayed teething, late walking, and knock-knee gait [9]. Without appropriate instructions on dosing and administration, a significant risk of vitamin D toxicity exists [13, 18]. The Endocrine Society states that vitamin D toxicity is defined as 25(OH)D concentrations exceeding 375 nmol/L [19], with levels above this toxic threshold associated with the onset of hypercalcemia [19–21]. Signs and symptoms of vitamin D intoxication are directly related to the effects of hypercalcemia [17, 20, 22], including decreased oral intake, nausea, vomiting, constipation, weakness, lethargy, and generalized malaise [17, 20, 21]. Hypercalcemia and vitamin D toxicity can lead to serious complications including renal failure, hypertension, and nephrocalcinosis [17, 23, 24]. Medullary nephrocalcinosis can be detected by ultrasound [9]. Nephrocalcinosis is a common pathological condition, characterized by hypercalciuria and/or hypercalcemia, with only 10% of cases caused by vitamin D intoxication [9]. Treatment for vitamin D toxicity usually involves removal of the exogenous source, intravenous fluid hydration, diuretics, a low-calcium diet, the administration of steroids, bisphosphonate therapy, and sometimes, renal dialysis [6, 23–25].

## 4. Conclusions and Recommendations

With clear verbal and written instructions for the dosage and administration of vitamin D supplements, as well as clear warnings of the potential risks of overdose, vitamin D intoxication could be an easily avoidable condition. Parents should also be advised to carefully read the instructions provided for any over-the-counter vitamin D supplements. Moreover, the inclusion of proper medication reconciliation with each visit to a pediatric clinic could help parents to avoid this type of mistake.

## Figures and Tables

**Table 1:** Summary of daily laboratory results during patient's hospital stay.

Day/lab.	Ca (mmol/L), NR: 2.25–2.75	PO_4_ (mmol/L), NR: 0.81–2.26	BUN (mmol/L), NR: 1.8–6.4	Cr (umol/L), NR: 11–34	Urine Ca : Cr ratio, NR: 0.08–0.57
Day 1	5.60	1.28	5.9	32	6.17
Day 2	4.69	1.23	4.8	28	9.83
Day 3	4.42	1.05	2.9	29	—
Day 4	4.17	1.28	2.2	29	—
Day 5	4.33	1.22	1.9	33	2.58
Day 6	3.92	1.20	1.6	30	—
Day 7	4.19	1.30	2.6	31	—
Day 8	4.14	1.12	2.8	31	—
Day 9	3.44	0.85	1.0	24	2.71
Day 10	2.96	0.84	<0.8	26	—

^*∗*^NR, normal range.
